# Overwriting the past with supervised plasticity

**DOI:** 10.7554/eLife.76320

**Published:** 2022-01-20

**Authors:** Xingyun Wang, Richard Naud

**Affiliations:** 1 Department of Cellular and Molecular Medicine and the Brain and Mind Research Institute, University of Ottawa Ottawa Canada

**Keywords:** plasticity, learning, place cell, dendrites, computational model, hippocampus, Mouse

## Abstract

Triggered activity bursts in place cells can increase and decrease the strength of some inputs.

**Related research article** Milstein AD, Li Y, Bittner KC, Grienberger C, Soltesz I, Magee JC, Romani S. 2021. Bidirectional synaptic plasticity rapidly modifies hippocampal representations. *eLife* **10**:e73046. doi: 10.7554/eLife.73046

Our ability to retain and remember information has fascinated humans as far back as classical antiquity. Already Cicero mused about the formation of memory, “*For, what is it that enables us to remember?*” (…) *Do we think there is* (...) *a sort of roominess into which the things we remember can be poured as if in a kind of vessel?* (...) *Or do we think that memory consists of the traces of things registered in the mind?*” (Stewart, 2009). Two thousand and eighty years later, having a tangible substrate for the physical basis of memory and unprecedented access to neurons during learning, these questions remain relevant.

When the brain forms memories or learns a new task, it encodes the new input by tuning the connections between neurons. These synaptic connections can be strengthened or weakened in a process called plasticity, which is a key part of learning new skills. It is thought that synaptic signaling can be amped up or dialed down to change the output of the connection between two neurons, but exactly how this happens remains unclear. Is it directed by a supervisory signal, or does it solely rely on the affected neurons?

Certain factors modulating synaptic plasticity have been well established (Roelfsema and Holtmaat, 2018; Magee and Grienberger, 2020). Recent studies have uncovered a potentially new mechanism that regulates synaptic plasticity in the hippocampus (Bittner et al., 2015; Bittner et al., 2017). There, complex bursts of activity called plateau potentials increase the strength of synapses that have been stimulated in the past few seconds. Crucially, the nature of these potentials indicates that they are triggered by signals from other parts of the brain.

Plateau potentials act on so called place cells in the hippocampus, which are important for navigation and spatial memory. Plateau potentials can increase the strength of recently stimulated synapses and can abruptly activate otherwise silent place cells when the body is in a specific location. However, it was not clear if they would also be able to decrease the strength of synapses, which would enable the brain to fine-tune the connectivity of synapses and the animal’s behavior. This way, the brain could update its map of the environment by causing active neurons to stop responding at one location and instead respond at a new position.

Now, in eLife, Jeffrey Magee, Sandro Romani and colleagues at Stanford University School of Medicine, Rutgers University, Baylor College of Medicine and Janelia Research Campus – including Aaron Milstein as first author – report new insights into plateau potentials (Milstein et al., 2021). The team recorded place cells in the hippocampus of mice while the animals navigated a virtual environment. Then, they experimentally induced plateau potentials in these cells. This revealed that plateau potentials enable place cells to change which location in the environment they respond to by either increasing or decreasing synaptic strength.

Similar to previous studies, inducing a plateau potential in cells that did not previously encode a particular place caused that cell to encode the location of an animal at the moment of stimulation (Bittner et al., 2017). However, Milstein et al. go on to show that a plateau potential can cause a place cell to respond less at the location it previously encoded, and more at the new location at the moment of stimulation. This indicates that it is possible to overwrite what had been previously learned and that plateau potentials may serve as some ‘supervisory’ signal to instruct synapses.

Further experiments led the team to propose and then test a computational model in which a synapse being strengthened or weakened depends on how strong that connection was to start with (Figure 1). According to the simulations, a weak synapse exposed to a plateau potential can only become stronger. However, a strong synapse can also weaken – whether it does depends on the time between receiving the plateau potential and the synapse being active.

**Figure 1.**
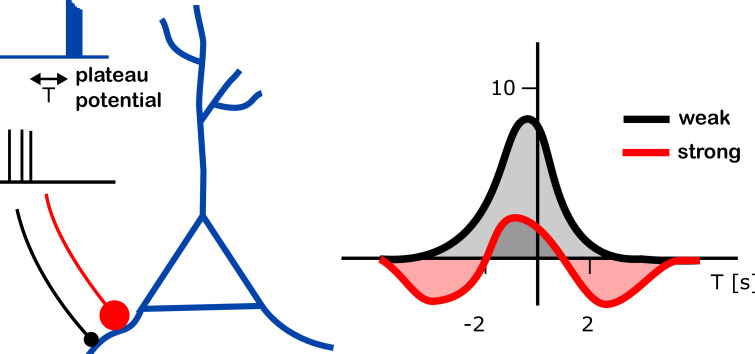
Changes in input strength induced by plateau potentials in place cells. A place cell (blue branched structure) is being stimulated to generate a plateau potential (blue trace on top left). The plateau potential happens either before, during or after the neurons are active, providing the input to the place cell (black trace, middle left). These inputs arrive either through strong synapses (red line with a large red dot) or through weak ones (black line with black dot). The plateau potential induced a change in strength of the input onto these place cells (right). The strenght of the input (vertical axis on right plot; units of mV) was found to depend on the relative timing of the plateau potential and the pre-synaptic activity (T; horizontal axis; units of s – T = 2s means that the plateau potential happened 2 seconds after the pre-synaptic activity). Different dependencies were found in weak (black) and strong (red) connections (right panel). Whereas an increase of input strength was typically observed (up to 8 mV at T = 0; gray shaded area), a decrease in input strength (by about 2 mV at T = 3 s or T = –4 s; red shaded areas) was observed for strong synapses at intermediate timing (T is between -5s and -2s, or between 2s and 5s).

Milstein et al. successfully demonstrate an additional mechanism of synaptic plasticity that enables rapid and reversible learning. It remains to be seen if this type of ‘supervised’ plasticity is consistent with other forms of plasticity observed in different regions of the brain, such as the cerebellum or cortex (Albus, 1971; Marr and Thach, 1991; Ito, 2008; Magee and Grienberger, 2020). Also, precisely how does the supervisory signal originate? And is supervised plasticity sufficient to explain the learning of complex tasks (Payeur et al., 2021), as well as the formation of episodic memory?

We may see a parallel between Cicero’s mechanism of pouring and today’s supervised plasticity, and between his “*trace of things registered*” and today’s connection strengths. The synapses (“trace of things registered”) are created by previous learning events, which form a network of varying strength (the “vessel”) on which the plateau potentials (and through them, the new life events) can act (be “poured”). It may well be that both processes are interlinked, and that learning – and memory – might both arise from these processes.
